# Empowerment strategies of the Mentor Mother peer support program among mothers who have migrated to Sweden: a photovoice study

**DOI:** 10.1186/s12889-024-19442-5

**Published:** 2024-07-16

**Authors:** Per Kåks, Mats Målqvist, Mark Tomlinson, Linnea Stansert Katzen

**Affiliations:** 1 Department of Women’s and Children’s Health, Uppsala University, Uppsala, SE-75 185 Sweden; 2 School of Nursing and Midwifery, Queen’s University, Belfast, United Kingdom; 3 Institute for Life Course Health Research, Department of Global Health, Faculty of Medicine and Health Sciences, Stellenbosch University, Stellenbosch, South Africa

**Keywords:** Social innovation, Peer support, Integration, Immigration, Migration, Parents, Children, Empowerment

## Abstract

**Introduction:**

A peer support intervention using ‘Mentor Mothers’ was implemented for mothers who had migrated to Sweden, living in socially disadvantaged communities. The Mentor Mothers had a high degree of freedom to develop strategies for facilitating empowerment of their clients according to perceived needs. This study aimed to investigate which empowerment facilitation strategies that Mentor Mothers perceived to be relevant, feasible and effective.

**Methods:**

Photovoice was used to generate qualitative data. Participants took photographs of their work which were then discussed during a focus group discussion and six individual semi-structured interviews. Data were analysed using thematic analysis.

**Results:**

Four overarching strategies to facilitate empowerment were identified, corresponding to distinctive perceived needs in the target group: (1) Informative support responded to a need for making sense of the external context, by helping mothers navigate society, the process of parenthood and cultural parenting norms. (2) Practical support addressed a need for managing challenges in daily life, by facilitating contacts with welfare services and authorities and to enhance parenting practices. (3) Psychosocial support addressed a need for improved mental wellbeing, by instilling feelings of safety and security in daily life, relationships and in contacts with public institutions. (4) Motivational support responded to a need for finding fulfilling purpose, by promoting social interaction, encouraging civic engagement and sharing the challenges and successes of others to inspire hope.

**Conclusions:**

These results highlight various aspects of peer support for empowerment facilitation that future interventions targeting immigrant parents can use in their intervention design.

## Introduction and aim

Despite a well-established welfare system, Sweden has persistent inequalities in health, including the health of young children [1]. In general, the inequalities that exist follow a social gradient, with adverse health outcomes correlating with both socioeconomic status and history of migration at the individual level [2]. This mirrors trends in Europe overall [3].

Part of this social gradient can be attributed to how different groups access the range of both preventive and curative healthcare and welfare services. In Europe, people who have immigrated from other regions of the world overall utilise preventative services to a lesser extent than the majority population [3]. This can be explained by a variety of factors, including language barriers, discrimination, stigma associated with ill health, low trust in social institutions and difficulties in gaining an overview of the range of services and support available [3–7].

Migration involves complex processes that can affect several categories of social determinants of health. This involves, for example, loss of social capital, changes in social status and reduced opportunities to assert one’s rights against public institutions [8, 9]. One type of approach to address migration-related social determinants of health is peer support to facilitate empowerment processes [10]. *Empowerment* is a wellness-focused concept based on the idea of promoting individual agency and self-efficacy through changing attitudes, knowledge and skills [11]. An important aspect of this concept is that people cannot be empowered by others – they can only empower themselves by gaining more of the various forms of power [12]. The role of others in empowerment can be to facilitate the process by creating the conditions for empowerment to take place. The concept differs from *resilience* in that empowerment aims at external change, enacted socially, while resilience aims at adaptations to withstand a situation as it is [11]. *Peer support* is a concept based on the idea that individual support is provided by someone who shares certain characteristics or experiences with the recipients [13]. It is close to the idea of facilitating empowerment as it is based on the notion that the support provider and the recipient share an understanding of both the existing concern, the possibilities to deal with it and what changes are perceived as meaningful. To reduce current gaps in the health of parents and children between social groups, a variety of parental support models based on peer support have been implemented alongside traditional health and welfare services in high-income countries [14].

One advantage of peer support for parents is that such programs can provide support with components that are difficult to include in professional services, such as deep emotional engagement and being a role model [15]. In cases where the delivering party belongs to the nonprofit sector, innovative service models are not limited by the rigidity that often characterises the public sector, where funding is often limited to solutions with proven effectiveness [16]. In other words, the nonprofit sector has a greater flexibility to test innovative intervention models and new strategies to achieve desired outcomes, and to adapt these according to what is perceived as valuable and appropriate.

### The mentor mother intervention

Since 2013, the non-governmental organisation *Tidigt föräldrastöd* (‘Early parental support’) has operated in socially disadvantaged areas in Gothenburg city, Sweden. The organisation employs doulas to provide emotional and administrative support before and during childbirth to mothers who have migrated to Sweden. These are mothers who have migrated to Sweden from countries outside of Europe and who are either newly arrived or who are in the early stages of becoming established in Sweden. In 2021, the doula support was broadened to include five peer supporters called Mentor Mothers (MMs). The aim was to build on the doula activity by providing support from birth until the children are five years old. All MMs are themselves mothers with a history of immigration and successful integration into Swedish society. They are all trained as doulas and as “cultural interpreters” and represent the largest language groups amongst their target population. MMs also typically live in the same area as their target group, and have varying levels of education prior to being trained as doulas and MMs. Their clients are predominantly mothers, but they occasionally provide support for fathers as well. Clients are either newly arrived in Sweden, or are in the process of becoming established in Sweden and have children that are under the age of 5. Clients are from various geographical, cultural, and language backgrounds, and all live in the geographical area covered by the MM intervention. The MM intervention is coordinated by a project coordinator.

The Mentor Mother project in Sweden was inspired by the work of the South African non-governmental organisation *Philani Maternal*,* Child Health and Nutrition Trust*. In 2002, Philani initiated a program with Mentor Mothers as a new approach to community outreach based on home visits by paraprofessional community health workers. The mentoring component, defined as continuity of relationship, being a role model and holistic support to clients, is a distinctive characteristic of the program [17]. This South African program has been extensively evaluated and has been shown to improve a number of maternal and child health outcomes, including prevention of vertical HIV transmission, breastfeeding practices and prevalence of child malnutrition [18–20].

In the Swedish setting, the MMs operate in the context of a health and welfare system that has high availability but not always high accessibility, prompting a need for strategies to facilitate the navigation of these systems. Each MM covers a specific language group and is individually responsible for a set of client mothers. The project plan states that peer support will be used to promote trust in and use of health and social welfare services as well as preschools [21]. Apart from this, the specific activities and methods used in the intervention are not strictly defined [21].

The MMs meet the client mothers individually, identify their social and health needs and address these needs either directly or by referring the client mothers to relevant health or welfare services. The individual meetings can take the form of home visits or meetings in other locations preferred by the client mother, and the frequency of these are adapted to individual needs. The MMs also holds regular courses with its group of client mothers on topics related to maternal and child health and social integration. The educational components of both individual meetings and group activities cover topics such as breastfeeding, nutrition, mental health, parent-child bonding, dental health, and how to navigate the Swedish welfare system.

The flexible description of the project gives the MMs the freedom to develop and adapt intervention content, methods and strategies. This is done according to both lived experience, professional expertise and empirical insights into what works, and what does not work with clients. By understanding what happens in this continuously developed and adapted intervention development, the MMs’ own expertise of the challenges and opportunities within the community can inform future development of peer support interventions.

Community empowerment can take place both on an individual and an organisational level, and it can include broader social and political changes [22]. Many of the barriers faced by the MMs’ clientele are structural issues such as discrimination and racism [23, 24]. While addressing such issues is essential to ensure social justice and the possibilities of equal life conditions, the intervention’s focus on the individual means that the approaches developed have prioritised the promotion of personal agency. The emphasis in this study is therefore on the needs of client mothers that can be addressed through support at the individual level.

### Aim

This study aims to identify MMs’ perceptions of which strategies to facilitate empowerment are relevant, feasible and effective in meeting the needs they perceive amongst immigrant mothers in socially disadvantaged areas.

## Methods

### Study design

This study followed a qualitative descriptive design and was conducted between April and December 2023. The data was generated using Photovoice, a method developed in 1994 by Wang and Burris as a way of promoting co-creation of knowledge in research settings that are otherwise characterised by power discrepancies [25]. Photovoice seeks to promote the democratisation of knowledge development and address some of the problems of traditional academic research involving vulnerable groups [26]. This is done by grounding the research in lived experience and promoting a sense of collective ownership of the problem formulation [26, 27].

The method builds on the idea that visual imagery can be an effective starting point for communicating both individual and community strengths and concerns. It allows for critical reflection on important topics, based on the research participants’ own framing of problems [28]. In Photovoice studies, the research participants are actively involved in how the generation of qualitative data takes place and what questions are asked during this process. This is achieved by collecting narrative data based on discussions about photos provided by the participants themselves. The existing literature on the use of participatory visual methods with community health workers suggests that it is a viable way to encourage reflection, understand complex health practices and identify key factors affecting their work [29]. In order to promote a diversity of reflections on individual photos and provide a fuller picture of community issues through collective interpretation, data collection in Photovoice studies often takes the form of focus group discussions or workshops [30].

### Data collection

A total population sample was used, with all MMs (*n* = 5) and their project coordinator (*n* = 1) employed by the implementing organisation participating in the study. The Mentor Mothers are the direct implementers of the intervention, while the project coordinator has a more managerial and coordinating role. For this sub-study, we focus on the implementation of the intervention, hence learning from the experiences of the Mentor Mothers and the project coordinator was the most relevant. In a different sub-study, client mothers (project participants) have also been interviewed. The MMs were provided with instructions during an in-person meeting to take photographs representing the forces that shape their clients’ needs and how these were met in their own work as MMs. During the meeting, the MMs had the opportunity to ask questions about the study, its methods and their own role as co-producers of knowledge. As the MM’s work varied from week to week, a time period of six weeks for photography was considered to be sufficient to document a wide range of situations where different empowerment strategies were applied. During the course of these weeks, the MMs took photos of relevant situations using their own phones, and each MM was then tasked with selecting a few photos that they felt were the most significant. All participants (*n* = 6) took part in a focus group discussion where the photos were discussed. These discussions were facilitated by the use of ‘SHOWeD’, a technique in which the discussions around each photo are guided by five questions:

1. What do you SEE here?
2. What is really HAPPENING here?
3. How does this relate to OUR lives?
4. WHY does this situation, concern, or strength exist?
5. What can we DO about it [30]?

The purpose of using these questions is to ‘identify the problem or asset, critically discuss the roots of the situation, and develop strategies for improving the situation’ [30]. The SHOWeD technique has been shown to be a valuable tool to move from superficial discussions to understanding deeper aspects of the captured phenomena [26]. In this study SHOWeD was used as a starting point for discussion around each photo, which then led to more unstructured and informal discussions. The focus group discussion lasted 153 min and involved discussions of 13 photos. Two of these photos were images of digital meetings, which the MMs included on their own initiative as this represented an important part of their work.

To provide an opportunity to explore topics that were difficult to address in a group setting, individual semi-structured interviews were also held with all participants (*n* = 6) directly after the focus group discussion. These interviews allowed for individual reflection on contextual barriers and facilitating factors, including factors in the internal context, i.e. the implementing organisation. Although the use of group discussions is the basis of the Photovoice methodology, triangulation of data through multiple data collection methods has been common in previous photovoice studies [31]. The interviews lasted 38–61 min. Both the interviews and the focus group discussion were conducted by the first and last author, were audio recorded and transcribed verbatim.

### Analysis

The transcripts were imported into ATLAS.ti 23.0 software for data management and analysis. The data were analysed thematically according to the methods described by Braun and Clarke [32]. This was done inductively applying a realist perspective, under the assumption that the MMs described the actual strategies they used, while a constructivist perspective also took into account that the issues the MMs were aiming to address reflected their own socially constructed realities. LSK and PK read through all transcripts before coding. One of the interview transcripts was then coded by both LSK and PK and codes were compared to enable agreement on a coding structure. This coding structure was used to code the remaining transcripts individually. The final coding was checked against the transcripts by LSK and PK to ensure its credibility. The codes were grouped and condensed into themes which were then discussed with all authors. The results were also discussed with study participants during a member check session involving all study participants to gather feedback on how they had been conceptualised and presented. This feedback was used to further refine the presentation of the findings.

### Methodological considerations

A relevant issue to raise in relation to the use of visual media in data generation is that there are limits to what is visually observable, and that Photovoice focus group discussions are shaped accordingly. In this study, the data collection involved discussions that went far beyond what was visually observable in the photos. However, it is worth reflecting on how visual imagery can steer discussions towards certain topics while steering them away from others.

In the classical Photovoice approach, participants are involved in data analysis by codifying and thematizing issues from the discussions. However, in this study, we opted for a researcher-led thematic analysis of the transcripts, similar to approaches used in some other Photovoice studies in the field [33, 34]. Participants were then involved in discussions around these researcher-generated themes through a member check session. This approach was chosen as the data was triangulated from the focus group and individual interviews and to enable theorization of described phenomena.

### Reflexivity

The focus group discussion and interviews were conducted by LSK and PK, whose backgrounds and experiences significantly shape their approach to this research. LSK, a white female postdoctoral researcher in global public health, has extensive experience working with the Mentor Mother program in South Africa, both as a programme manager and as a researcher. PK is a white male medical doctor conducting doctoral studies on the Mentor Mother program in Sweden, and has experience conducting research with socially disadvantaged migrant groups in other parts of Sweden. Their respective backgrounds, which include privileges and biases inherent to their cultural and professional contexts, influence how they formulate research questions, interact with participants, and interpret findings. While their positions of relative privilege and PK’s male identity could have introduced power dynamics affecting participant comfort and openness, both researchers had spent considerable time developing rapport with the Mentor Mothers prior to the study, fostering a more trusting and open environment.

## Results

Following six weeks of photographing, participants selected 13 photographs representing various aspects of their work that they saw as important. The discussions about the photographs and the subsequent individual interviews revealed a wide range of strategies that the participants themselves perceived as relevant, feasible and effective in their efforts to facilitate empowerment of individual clients. These empowerment strategies could be divided into nine sub-themes, following four overarching themes: informative support, practical support, psychosocial support and motivational support (Table 1).

**Table 1:** Themes, sub-themes and examples of codes

Theme	Sub-theme	Examples of codes
Informative support to meet a need for making sense of the external context	Providing a roadmap to navigating society	*Increasing understanding of benefits of health services* *Need for understanding the purpose of social services*
	Clarifying the expected process of becoming a parent	*Informing on what can be expected during delivery* *Need for knowledge of child health*
	Explaining cultural norms and expectations on parents	*Providing information on rights and obligations as parents* *Need for understanding parenting norms*
Practical support to meet a need for managing challenges in daily life	Facilitating contacts with the welfare system and authorities	*Booking appointments for healthcare* *Cultural misunderstandings in contact with authorities*
	Strengthening the parenting role through practical strategies	*Providing strategies for boundary setting* *Parents losing their parent role*
Psychosocial support to meet a need for improved mental wellbeing	Providing a sense of safety through togetherness	*Providing psychosocial support during divorces* *Poor maternal mental health*
Motivational support to meet a need for finding fulfilling purpose	Fostering a sense of purpose through increased social interactions	*Breaking isolation* *Need for a sense of belonging*
	Promoting civic engagement	*Encouraging mothers to vote* *Lack of trust in the political system*
	Inspiring hope and motivation by sharing the life stories of others	*Finding inspiration in others’ journeys* *Sense of missing a role in life*

Informative support to meet a need for making sense of the external context.

### Providing a roadmap to navigating society

A number of MMs took photos of work situations where they delivered educational initiatives relating to the organisation of Swedish society and the health and welfare system. The discussions around these photos highlighted how MMs perceived the need for strengthening the ability to understand and navigate Swedish society, and how this was perceived as empowering. This need for support consisted partly in an understanding of the functions of various institutions and the possibilities for different types of support that welfare services could offer. Such institutions included the social services and various health services to which client mothers could turn for support, as well as the political system whose organisation and structure were not obvious to everyone.

*There are a lot of links between rights and yes*,* the political process and votes and all that. Basic information about the political process. [Study participant #1]*

MMs and their coordinator described how a lack of understanding of societal services had led to mistrust in the services they offer, particularly with regards to social services. By increasing the knowledge about how these services were organised and operated, this mistrust could to some extent be mitigated.

*They were thinking that social services would come and take the children. They didn’t know at all about this preventive part. Now they get a lot of information*,* but little information at a time many times. [Study participant #1]*

The empowerment facilitation strategies also consisted of promoting an understanding of the benefits of using different types of public services, such as routine visits to childcare centres, dental appointments, vaccinations, parental support from the social services, and enrollment in preschools.

*[F]or example*,* dentists*,* they think that dentists are unnecessary for small children*,* they don’t exist in our countries*,* there are no dentists who start when the children are small. Why do they make appointments all the time? My son or my daughter has no teeth! And then she doesn’t come to this appointment*,* she doesn’t come to that appointment*,* they have to file a report of concern [to the social services]. That’s why we manage*,* we work with dentists*,* child health centres*,* we try and think together. Home visits are very good and very important. [Study participant #5]*

### Clarifying the expected process of becoming a parent

**Fig. 1:**
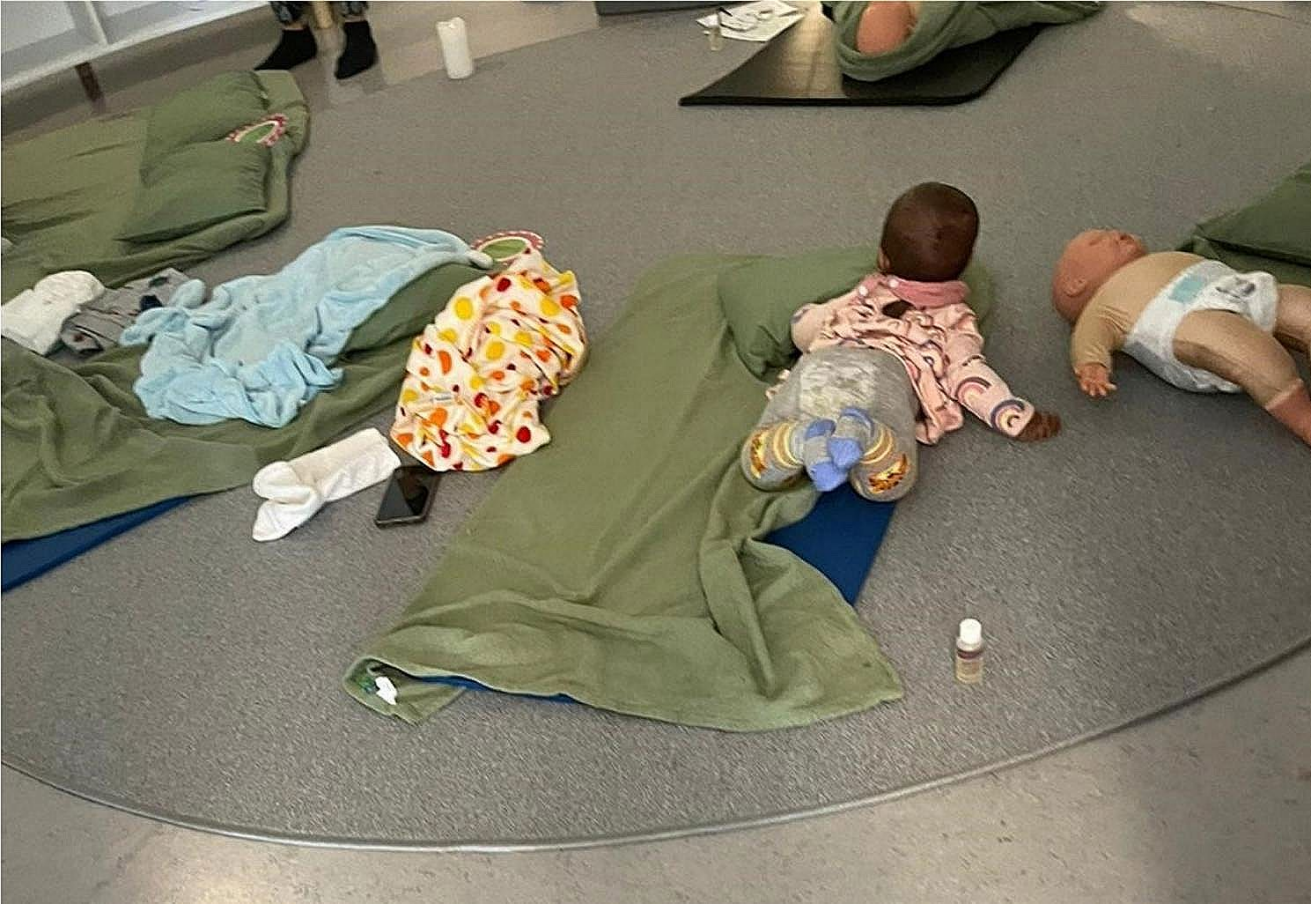
Group course on childcare

Another need that was repeatedly described by the MMs was a clearer understanding among expectant parents regarding what to expect during pregnancy, delivery and after delivery (Fig. 1). Meeting this need consisted of providing information on physiology and psychology, such as how a pregnant woman’s body is affected by hormonal changes, what postpartum depression is and what to expect when starting to breastfeed. The participants also actively tried to involve fathers by promoting their understanding of the process of pregnancy, childbirth, and the time after, and how they could support their partner if needed. The involvement of fathers as a strategy was also described as involving questioning normative distribution of responsibilities.

*[I] also try to inform him that there are changes in the anatomy and physiology of the mother when she is pregnant. There is an increase in prolactin and progesterone and oestrogen. It’s a war in there. There are changes in… Yes*,* and the baby blues*,* you could say*,* postpartum depression*,* many women fall into that. When they get information and the father gets information*,* they can try to support the woman*,* support the woman after the birth*,* when she’s breastfeeding she becomes very sensitive and she cries*,* and that makes… The prolactin hormone that is raised makes you depressed as well*,* and you get anxiety as well. [Study participant #1]*

### Explaining cultural norms and expectations on parents

The study participants described how many client mothers expressed that it was difficult to understand what was expected of parents in Sweden. This concerned both the rights and obligations that parents had towards their children, as well as the rights and obligations they had towards Swedish authorities and the health care system. By clarifying these expectations, the MMs described that they felt they could support their clients in their role as parents. The MMs described how many parents they met were afraid that setting limits for their children would be outside the norm of how parents can behave in the Swedish context. This, in turn, was described as leading to social services intervening in the family if the parents exceeded their rights. By clarifying parents’ rights and obligations, the MMs described how they could contribute to a better understanding of how parents were expected to handle different types of situations that could arise within the family.

*There is a huge difference between the way people raise children in their home country and here in Sweden. And this course sets out the basics*,* that you should give love to the children and create a good relationship between parents and children. [Study participant #3]*

### Practical support to meet a need for managing challenges in daily life

#### Facilitating contacts with the welfare system and authorities

**Fig. 2:**
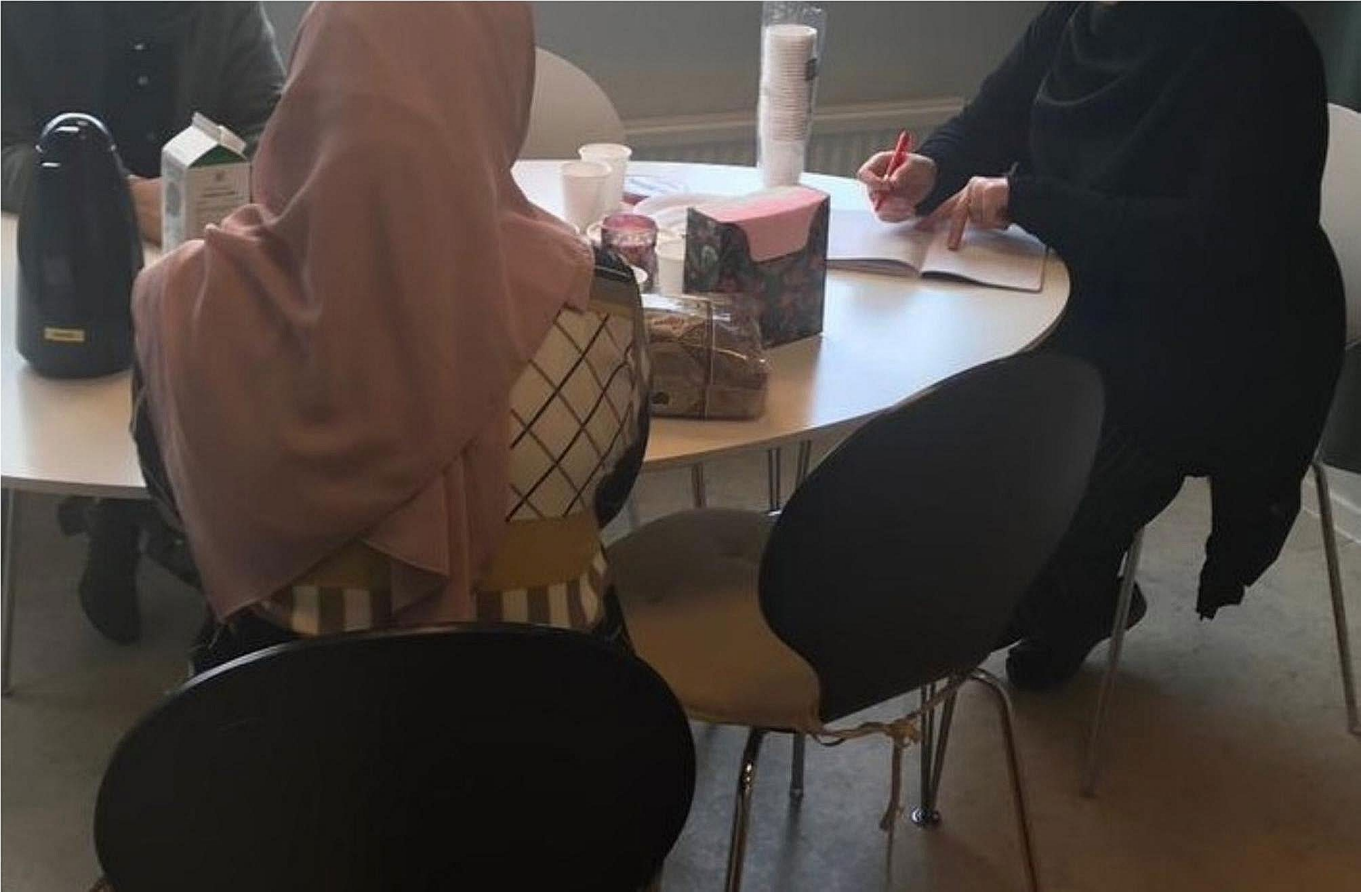
Mentor mother providing individual support to two client mothers

One concrete type of support that the MMs and their supervisors described was the importance of facilitating encounters with authorities and public services (Fig. 2). This consisted partly of MMs acting as interpreters during in-person or telephone meetings, where the interpreting also involved a cultural interpretation component. This meant that the MMs not only interpreted word-for-word but also explained the cultural norms underpinning what was said. The facilitation of contacts also entailed explaining letters from authorities and healthcare providers, helping client mothers book appointments within the health service and helping them find their way to services providers and authorities by physically accompanying them to meetings.

This picture is of individual support with a family, or two mothers who have the same situation. And they need help. And we try to make an appointment. And I heard from them what concerns they have, in what way I should help them. I take their contact details and call authorities to help them. […] We have made an appointment, sit with them, talk and listen to them, try to help them in some way, try to call different authorities to find answers or solutions to their problems. [Study participant #5]

By facilitating these contacts with authorities and welfare services, the MMs described that their clients not only felt empowered in the moment, but also learned to handle these contacts more independently in the long run.

### Strengthening parenting through practical strategies

**Fig. 3:**
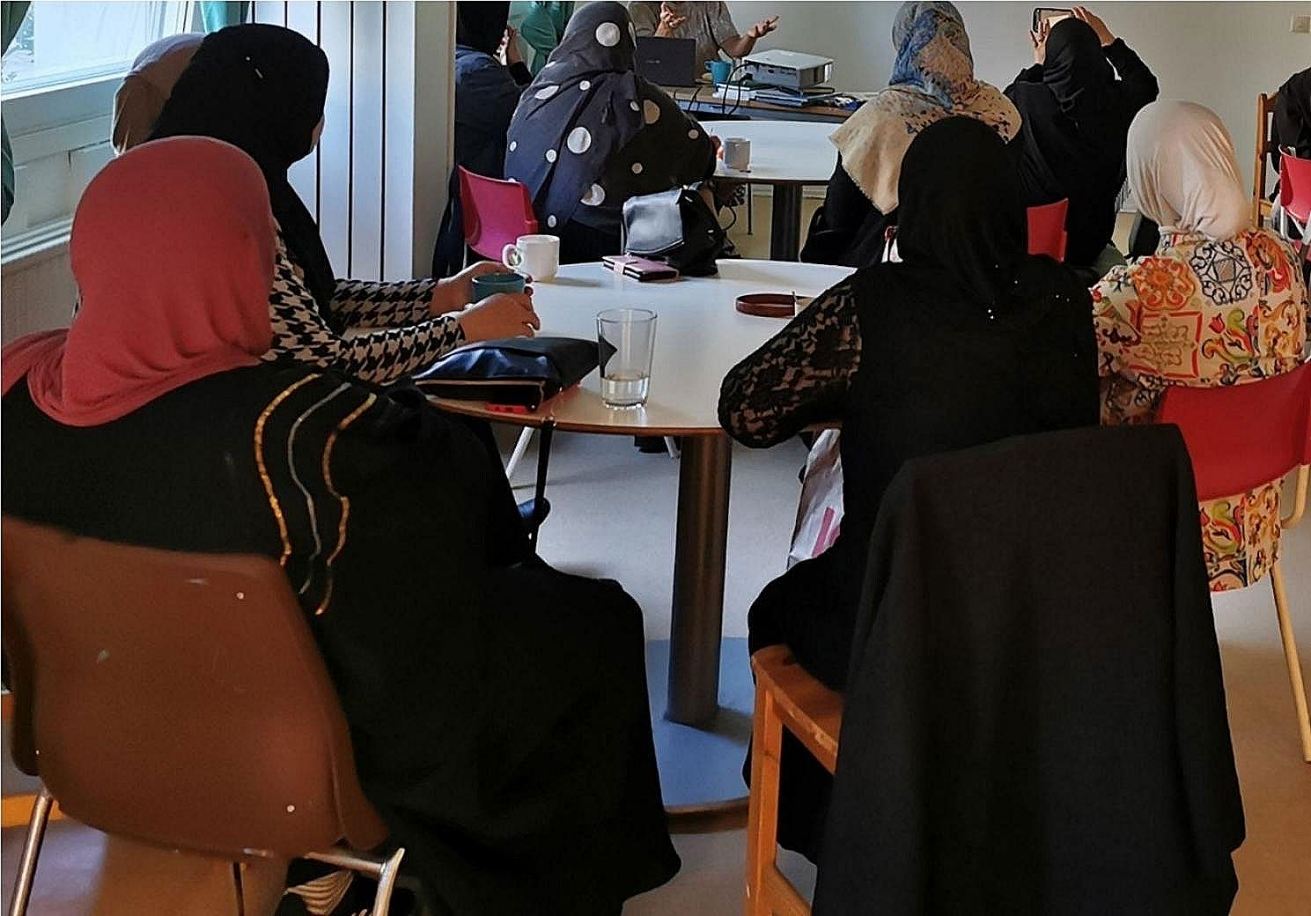
Group course on boundary setting within the family

A number of parents expressed to MMs that they had experienced a sense of having lost their parenting role. In response, through providing concrete strategies for family routines and boundary setting (Fig. 3), the MMs and their supervisor described how they could contribute to a clearer parental role for their clients. This was described as a key to reducing stress as parents and children were given structure in their daily lives and clear house rules to refer to, which contributed to the children’s interaction with others both inside and outside the family. The MMs described how they saw this as contributing to a sense of control in the everyday life of their clients.

[This photo] touches us very much, this particular course because one of the mothers in the picture, she came to us after the course ended and told us how her situation had changed at home. [W]hen she met this amazing preventive social worker, she got methods that she could use, that strengthened her parental role. And she also got methods for reducing stress, giving the children responsibility, participation, sitting and talking beforehand in preparation. And she also received information about what the law says and what your rights are as a parent. So when she took the method and used it at home, she came back and told her story about how it has changed. [Study participant #4]

### Psychosocial support to meet a need for improved mental wellbeing

#### Providing a sense of safety through togetherness

The psychosocial aspects of the intervention were discussed in terms of a sense of safety. By providing a sense of safety, the MMs stated that they could meet the need among the client mothers regarding sharing their psychosocial burden caused by external stressors. By creating a sense of togetherness and instilling a sense of safety, client mothers were able to speak more freely about their problems. This sense of safety was built up over time as the relationship between MMs and client mothers became stronger. They also stated that they contributed to feelings of security in meetings with authorities such as social services by participating in these together with their clients. This allowed client mothers to assert their rights in such situations. Another important psychosocial aspect of the intervention was described as providing psychological support to client mothers with difficult relationships, such as supporting women going through divorce or instilling a sense of safety and belonging in women who did not feel appreciated by their partners. It could also mean discussing topics such as self-esteem and autonomy and questioning partnership norms to promote the psychological aspects of women’s empowerment.

*There was a Somali mother who was reported for concern [to the social services]*,* [project manager] also knows about that mother*,* she was very scared and they sent the report to her home without explanation. […] We went home and sat with her*,* and she exhaled and talked. And that meeting was very important I think*,* for us who worked here*,* we mentor mothers. To just sit with them. We can’t do very much*,* but we can provide support and show them that we are here for you. We will help you*,* what do you want? You can breathe*,* it’s okay. You can move on… It’s not like they’re going to take your child right away. But the process is like this*,* and [mentor mother] will help you if you need help. And then the mom or the parents get a little bit stronger and calmer. [Study participant #3]*

### Motivational support to meet a need for finding fulfilling purpose

#### Fostering a sense of purpose through increased social interactions

To counteract social isolation and contribute to increased social capital, MMs discussed how they use both motivational efforts and a deliberate bringing together of individual participants. By motivating participants to seek and interact with others outside the home, MMs felt that they could contribute to both increased participation in the community and the expansion of clients’ social networks. This was also described as aiming to counteract mental distress and to promote language skills in Swedish among the client mothers. Some of MMs’ work took the form of group courses, and they described how they used these courses to ensure that participants got to know each other and had time to interact during the course sessions. Breaking the client mothers’ social isolation was described as a way to increase their willingness to invest time and energy in their quest for a better and healthier life for themselves and their family.

*It is important that they get to know each other. It’s important that they try to go out with small babies. Because in our culture*,* when you give birth you have to stay forty days or more at home*,* otherwise it’s not so good if the mother takes the children and goes out. We try to make them think that no*,* bring your child and come. [Study participant #2]*

### Promoting civic engagement

The participants in the study discussed client mothers’ involvement and participation in Swedish society as an important goal in their work, corresponding to a need for social integration. This was described as motivating client mothers to feel that they could change not only their own life situation but also the structures of society at large. To promote civic engagement, MMs and their coordinator described discussing with their clients the importance of, for example, voting in local and national elections. They also organised meetings between groups of client mothers and local politicians to stimulate a sense of proximity to those in power and an understanding of the ability to influence them.

*We provide so many participants with information about voting*,* but sometimes they feel that they don’t have confidence in anybody*,* or they think that they should do nothing for them. I’m not going to bring change if I vote*,* so they sit at home. But we try to explain to them that even if you have nothing*,* go and vote because it will not be against your interests*,* or your vote will go to something else. It’s better that you go and do it. [Study participant #2]*

### Inspiring hope and motivation by sharing the life stories of others

**Fig. 4:**
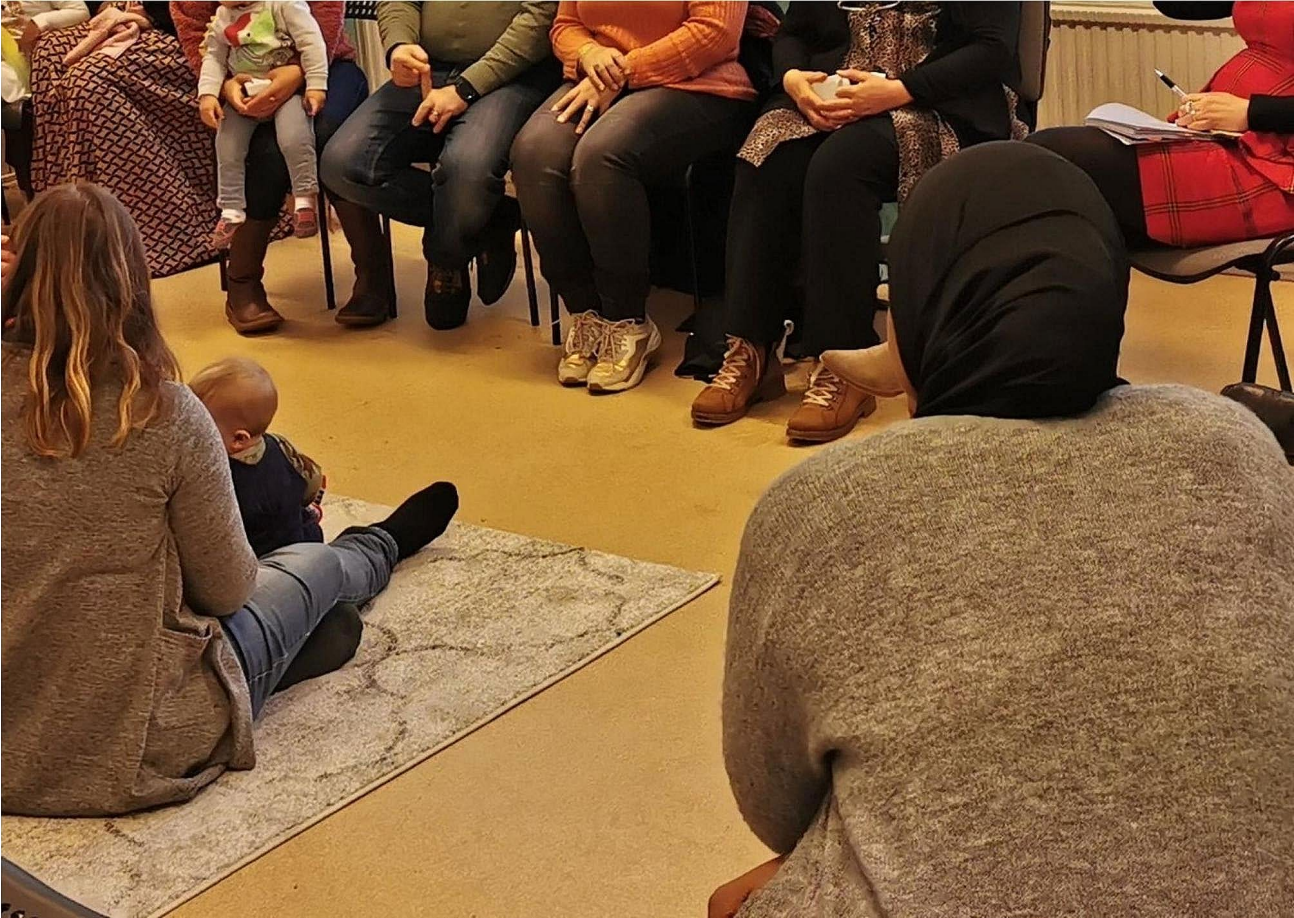
Group course on parenting practices

In response to a perceived need for motivating clients to invest time and energy in themselves and their families, an empowerment facilitation strategy was to show how they were not alone with their problems, and how other people had solved similar difficulties in the past. This could consist of MMs themselves describing to their clients how they had struggled earlier in life to create good conditions for themselves and their children, and how they had overcome problems. It could also include discussing difficulties in a group (Fig. 4), so that client mothers could normalise their problems together and inspire each other by sharing their life journeys. The challenges discussed in this way in groups could be both integration-related, such as social isolation or fear of authorities, and health-related, such as pregnancy or breastfeeding problems. MMs stated that by hearing the life stories of others, including how such challenges had been overcome, the client mothers could be strengthened in their sense of what they themselves were capable of.

*We all sit together. When she sits and talks*,* you can see that she has problems. Others*,* you know*,* women open up and she sees that it’s not just me who is [isolated] at home*,* it’s not just me who has problems. There are others too. You feel a bit*,* how can you say*,* you feel that it’s not just me. And that makes you feel that it’s less stressful. [Study participant #2]*

## Discussion

This study used Photovoice to explore which strategies for facilitating empowerment that paraprofessional peer supporters perceived as effective and feasible in their work with parents with experience of migration. The results of the qualitative analysis indicated that these strategies follow four overarching themes: informative support to meet a need for making sense of the external context, practical support to meet a need for managing challenges in daily life, psychosocial support to meet a need for improved mental wellbeing, and motivational support to meet a need for finding fulfilling purpose. These strategies are perceived as effective by the implementers of this intervention, and we argue that these strategies could also be effective in other interventions and geographical areas.

A previous WHO report on empowerment in health promotion has highlighted that the most effective strategies are those that reinforce participation to ensure autonomy in decision-making, sense of community, local bonding, and psychological empowerment [35]. In our analysis, the themes of informative and practical support can be interpreted to correspond to autonomy in decision-making, while motivational and psychosocial support correspond to a sense of community, local bonding and psychological empowerment.

Regarding empowerment as an outcome, previous research by Dickerson has outlined three constructs: a sense of personal competence, self-determination, and social engagement [36]. These constructs are in line with the strategies within the mentor mother intervention aiming to contribute to a greater knowledge base amongst the client parents, a promotion of their ability to assert their rights in society, and a greater social capital and participation in society. It also roughly corresponds to a nomological framework by Zimmerman, where psychological empowerment is constituted of an intrapersonal component (perceived competence and control), an interactional component (skill development and resource mobilisation) and a behavioural component (community involvement and participation) [37].

The empowerment facilitation strategies developed by the MMs can also be interpreted using the Sense of Coherence (SOC) model developed by Antonovsky [38]. SOC is a salutogenic model of factors determining how adversity is managed, aiming to explain why certain individuals remain healthy despite experiencing internal and external stressors. It is constituted by three domains.

The first domain is *comprehensibility*, referring to whether an individual’s life situation is perceived as structured, predictable and explicable. The MMs’ strategies aiming to provide informative support can be understood as an attempt to broaden the knowledge base that enables the client mothers to interpret and understand their own situation more comprehensively. This understanding consists partly of grasping the external context, such as how Swedish society is organised. It also includes their own situation as a new parent, including various aspects of childrens’ health and development and the expectations of parents in the Swedish context.

The second domain is *manageability*, which involves resources and capacities to meet the demands of stimuli and stressors. The MMs provided practical support in contacts with authorities and services, practical strategies for parenting such as boundary setting, and psychosocial support by instilling a sense of safety in challenging situations. These strategies are all based on an idea of facilitating the management of challenging circumstances.

The third domain is *meaningfulness*, which entails whether challenges are perceived to be worth investing engagement in dealing with. The MMs provided motivational support aimed at increasing social interactions, community participation and taking inspiration from the life journeys of others. The motivational elements of their strategies can all be linked to the idea of increasing clients’ willingness to engage, either in improving their own life situation or in contributing to a stronger society.

In summary, the empowerment facilitation strategies perceived as relevant, feasible and effective by the MMs and their supervisor corresponded to the three domains of SOC. Their strategies aimed to increase client mothers’ understanding of their own situation, their ability to manage difficult situations and circumstances and their willingness to invest time, energy and commitment to improve the situation for themselves and their families. Based on these conceptual similarities, it can be argued that the strategies used by the MMs to support their clients’ empowerment were perceived as effective in that they strengthened the three SOC dimensions, though this was not explicitly stated.

Empowerment-focused interventions have previously been found to increase SOC, which points to a link between the two concepts [39–41]. However, a survey study on mental health promotion in northern Norway has indicated through factor analysis that SOC and empowerment are separate concepts [42]. In the study, the two concepts had a partial overlap, where SOC as a whole corresponded to a factor consisting of power/powerlessness, but not other factors such as self-determination, self-esteem and community activism. This partial overlap could be understood as a reflection of Brodsky and Cataneo’s distinction between the concepts of empowerment and resilience [11]. The empowerment and resilience concepts are posited as converging in a response to adversity, but diverging in their focus on external, transformative or internal, adaptive enactment. This line of thought also mirrors how SOC has been conceptualised in later years as having both a perceptual and a behavioural component [43]. In other words, SOC might differ from empowerment by including an element of internally focused response to adversity, an aspect that was outside the scope of this study.

### Strengths and limitations

A strength of this study was the use of Photovoice, which was perceived by the authors as a method that enabled the collection of high-quality qualitative data. The study participants themselves had the opportunity to use pictures to formulate the starting points that were important in the data collection. This was perceived to provide depth in the workshop and interviews, as well as discussions that took shape based on what the participants themselves felt was important. The study participants’ deep understanding of the context, where they shared many characteristics and personal experiences with their clients, also contributed to the quality of the data collected.

In addition to encouraging people to reflect on their societies and promoting critical dialogue, Photovoice also aims to bring about change [30]. This is achieved by disseminating the results to those who have the ability to influence the concerns highlighted during the research process. As this study was conducted in an implementation science context, we chose to interpret this as meaning that change can be achieved through knowledge exchange between health researchers and knowledge users. The latter, in this case, consisted of people who were involved in the programme at an organisational level, who were presented with the findings at an early stage. The study, however, did not actively involve stakeholders at the policy level.

The small sample size limited the amount and variety of data. This is a limitation not only for this study, but for all types of research aimed at studying particularities in the form of small, localised phenomena. In the traditional use of the Photovoice method, a sample size of about seven to ten is recommended to allow for practical ease and in-depth discussion [30]. The number of participants in later Photovoice studies varies widely, but it is not rare to include fewer participants than in studies based on other qualitative methods [44]. Since a total population sample was used in this study, a larger sample was not an option in this case. Consequently, the results should be interpreted as an example of how strategies for facilitating empowerment can be developed in a specific context, rather than providing an exhaustive overview of this type of practice. This type of smaller study can add value in under-researched areas. In this case, the area is the bottom-up development of empowerment-focused interventions by the non-profit sector. Within this area, this study contributes to an understanding of how people with lived experience of social difficulties choose to support others going through the same processes, an aspect which we have not seen previous studies explore.

## Conclusions

This study aimed to investigate which strategies for facilitating empowerment that MMs perceive as relevant, effective and feasible to address the perceived needs amongst their clients, using Photovoice as a method for qualitative data generation. The subsequent thematic analysis identified four overarching strategies. Firstly, informative support was used to promote the ability to navigate the community, understand the process of becoming a parent and to recognize cultural norms and expectations around parenting. Secondly, practical support was provided to facilitate contacts with health and welfare services and public institutions, to enhance parenting practices. Thirdly, psychosocial support was given to instil feelings of safety and security in daily life, relationships and in contacts with public institutions, allowing clients to assert their rights. Fourthly, motivational support aimed to promote a sense of purpose through increased social interaction, encouraging civic engagement and sharing the challenges and successes of others to inspire hope. These results highlight both perceived needs amongst the mentor mother clients, and various aspects of peer support for supporting empowerment that future interventions can use in their intervention design.

## Data Availability

The datasets generated and analysed during the current study are not publicly available due to the absence of such an agreement with the study participants, but are available from the corresponding author on reasonable request.
